# Use of brachial plexus blockade and medetomidine-ketamine-isoflurane anaesthesia for repair of radio-ulna fracture in an adult cheetah (acinonyx jubatus)

**DOI:** 10.1186/s12917-014-0249-9

**Published:** 2014-10-10

**Authors:** Peter Kimeli, Eddy M Mogoa, Willy E Mwangi, Ambrose N Kipyegon, Gilbert Kirui, Daniel W Muasya, John D Mande, Edward Kariuki, Dominic Mijele

**Affiliations:** Department of Clinical Studies, Faculty of Veterinary Medicine, University of Nairobi, P.O. Box 29053–00625, Kangemi, Kenya; Kenya Wildlife Service, P.O. Box 40241–00100, Nairobi, Kenya

**Keywords:** Wild cats, Forelimb fracture, Anaesthesia protocol

## Abstract

**Background:**

Regional anaesthetic techniques have been used in combination with systemic analgesics during small animal surgery to provide multimodal analgesia. Brachial plexus nerves block using local anaesthetics provides analgesia of the thoracic limb through desensitization of the nerves that provide sensory and motor innervation. This has been shown to reduce intra-operative anesthetic requirements and provide postoperative pain relief. Decreasing the doses of general anaesthetics allows more stable cardiopulmonary function during anaesthesia and the development of less side effects. The present case reports a successful use of brachial plexus blockade to supplement medetomidine-ketamine-isoflurane anaesthesia for repair of radio-ulna fracture in an adult cheetah (*acinonyx jubatus*).

**Case presentation:**

An adult male Cheetah weighing about 65 kg was presented with a history of leg carrying lameness of the left forelimb sustained following a car accident a week earlier. Clinical examination under general anaesthesia revealed slight dehydration and a swelling with a wound on the caudo-medial aspect of the left radio-ulna region. Crepitation was present on manipulation and radiography confirmed a complete transverse radio-ulna fracture of the left forelimb, which required open reduction and internal fixation. Brachial plexus blockade using lignocaine hydrochloride was used to supplement medetomidine-ketamine-isoflurane anaesthesia for the surgical procedure. Isoflurane anaesthesia was maintained at 0.5 - 2.0% throughout the surgical procedure, which was uneventful. Temperature and cardio-pulmonary parameters remained stable intra-operatively. Limb paralysis extended for 5 hours post-operatively, suggesting prolonged anaesthesia.

**Conclusion:**

To the researchers’ knowledge, this is the first reported case of the use of brachial plexus blockade to supplement general anaesthesia to facilitate forelimb surgery in an adult cheetah. The use of brachial plexus block with a light plane of general anaesthesia proved to be successful. Brachial plexus block had a sparing effect on isoflurane anaesthesia as evidenced by the concentration used for maintenance of anaesthesia and the stability of the cardiopulmonary function. Moreover, absence of autonomic cardiopulmonary reactions to the surgical manipulation may be attributed to the efficacy of brachial plexus block. This anaesthesia protocol is therefore recommended for surgeries of the forelimb in wild cats.

## Background

Over the past several years, regional anesthetic techniques have been used in combination with systemic analgesics during small animal surgery to provide multimodal analgesia [1]. This has been shown to reduce intra-operative anaesthetic requirements and provide postoperative pain relief [2]. Decreasing the doses of general anaesthetics and other drugs may allow more stable cardiopulmonary function during anaesthesia and the development of less side effects [3].

Brachial plexus block using local anaesthetic drugs provides analgesia in animals undergoing surgery of the thoracic limb through desensitization of the nerves that provide sensory and motor innervation [2,4]. Three techniques of performing brachial nerve blockade have been described and these include; blind needle placement using anatomical landmarks, the use of peripheral nerve locator or nerve stimulator and ultrasound guided needle placement [3].

Lidocaine is the most frequently used local anesthetic solution for regional anaesthesia in veterinary practice [5] and it causes a blockage of the sensory and motor fibers. Regional anaesthesia of the brachial plexus with local anaesthetic drugs has been reported to provide an excellent means to control post-operative pain after forelimb surgery in animals [6].

The use of a brachial plexus block has been described in humans [7], dogs [4,8-10], cats [2] and sheep [6] in either clinical or experimental settings. This study reports the successful use of brachial plexus blockade using lidocaine in an adult cheetah under a light plane of medetomidine-ketamine-isoflurane anaesthesia during the management of a radio-ulna fracture.

## Case presentation

**Figure 1:**
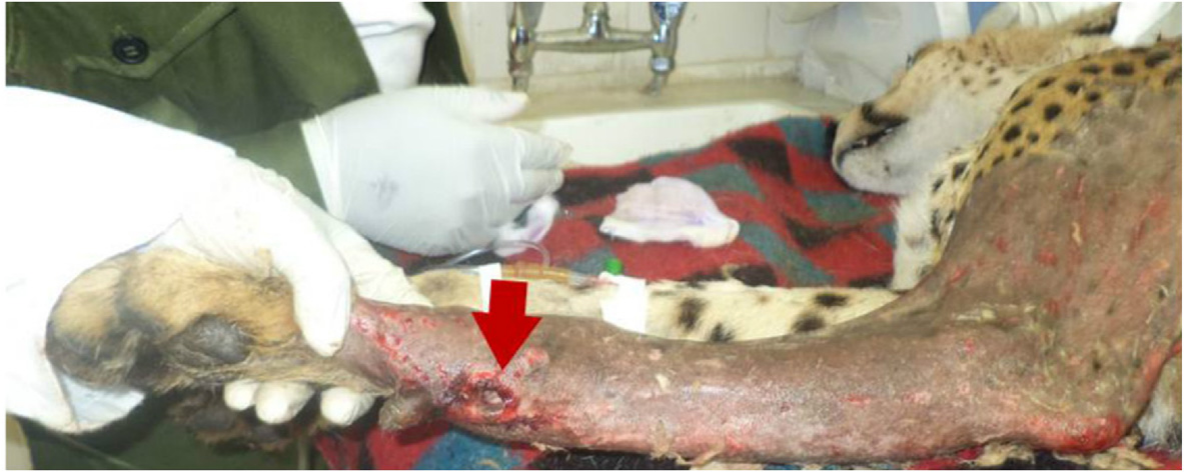
**Injured left forelimb.** Note the wound on the caudo-medial aspect of the antebrachium (arrow).

**Figure 2:**
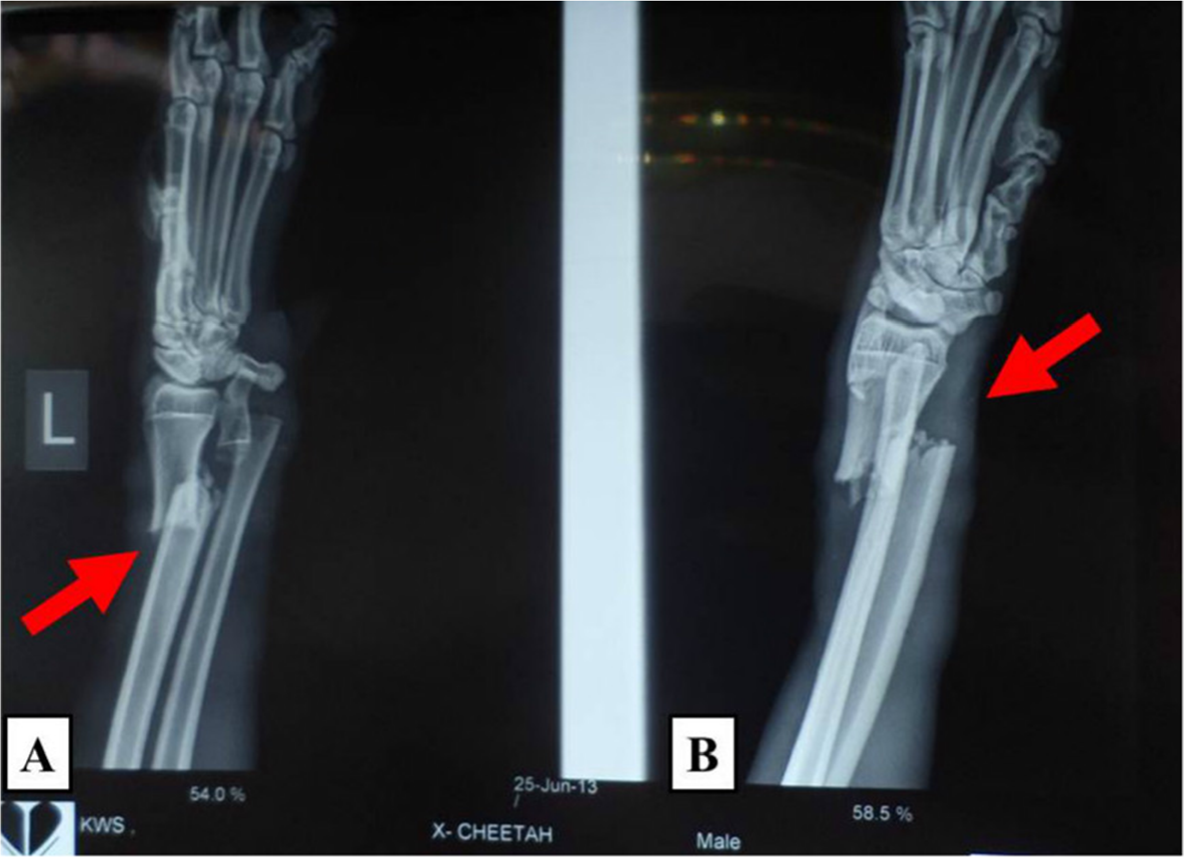
**Lateral (A) and Antero-posterior (B) radiographs of the left forelimb**. Note the complete transverse fracture of the distal radius and ulna (arrow).

An adult male Cheetah weighing about 65 kgs was presented to the Small animal Clinic, University of Nairobi with a history of leg carrying lameness of the left forelimb following a car accident near Tsavo National Park, Kenya, one week earlier. Clinical examination under general anaesthesia revealed slight dehydration and a swelling with a wound on the caudo-medial aspect of the left radio-ulna region (Figure 1). Crepitation was present on manipulation and radiography confirmed a complete transverse radio-ulna fracture (Figure 2) of the left forelimb. A decision was taken to manage the case surgically.

Anaesthesia was induced using 50 μg/Kg of Medetomidine Hcl^a^ and 5 mg/Kg of Ketamine Hcl^b^ combination by darting using a blow gun to allow pre-surgical preparation and endotracheal intubation. A blood sample was collected for hematology which revealed severe leucocytosis and slight lymphocytosis (Table 1).

**Table 1:** **Hematology results of a cheetah admitted to Small Animal Clinic, University of Nairobi, for management of complete Radio-ulna fracture**

**Hematological parameters**	**Hematological values**	**Normal range in domestic cats**
WBC (m/mm^3^)	42.66	5.0-18.0
Lym (%)	36.1	5.0-30.0
Mon (%)	3.7	2.0-6.0
Gra (%)	60.2	40-80
RBC (m/mm^3^)	5.09	4.0-9.0
MCV (fl)	52.4	35.5-55.0
Hct (%)	26.6	24.0-45.0
MCH (pg)	18.4	16.0-24.0
MCHC (g/dl)	35.3	28.0-40.0
RDW	13.7	8.0-12.0
Hb (g/dl)	9.4	9.5-15.0
THR (m/mm^3^)	145	120-500
MPV (fl)	9.4	4.0-7.0
Pct (%)	0.14	_
PDW	8.6	8.0-12.0

**Figure 3:**
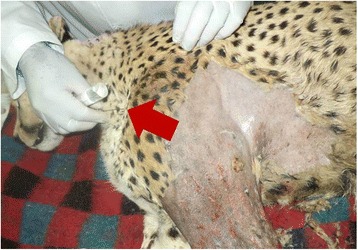
**Brachial plexus nerve block**. This was achieved through blind needle placement by introducing a hypodermic needle (arrow) through a point cranio-medial to the scapula-humeral joint and advanced in a caudo-dorsal direction.

After general anaesthesia, a brachial plexus block was achieved by infiltrating 20 ml of 2% lidocaine hydrochloride^c^ into the axillary area medial to the left shoulder joint. This was done by introducing a needle (16 gauge × 12 cm) through a point cranio-medial to the scapula-humeral joint and advanced toward the costochondral junction parallel to the vertebral column, while the patient was on right lateral recumbent position (Figure 3). On ascertaining that the needle tip was in the right position, the syringe was aspirated to ensure that the needle was not in a blood vessel and 15 ml of the local anaesthetic injected at the site slowly in a fan-shaped pattern and 5 ml injected as the needle was withdrawn. Complete analgesia of the blocked limb was achieved within 10 minutes of brachial plexus blockade as ascertained by complete loss of pedal reflex in the left forelimb, which was present in the contra-lateral limb, following clamping of interdigital skin. Anaesthesia was maintained with isoflurane^d^ in oxygen using a rebreathing anaesthetic machine. The maintenance vaporizer settings of isoflurane ranged between 0.5 and 2.0%, throughout the procedure. Anaesthetic depth was monitored with the help of palpebral reflex, pedal reflex, eye position and ear twitch reflex. 3000 mls of lactated Ringer’s solution was administered intravenously throughout the procedure. Long acting amoxicillin trihydrate^e^ at a dosage rate of 10mgkg^−1^ was administered 24 hours before surgery, immediately after surgery and on alternate days for four more times. Post surgical analgesia was achieved through a single injection of carprofen^f^ subcutaneously, at a dosage of 4 mg/kg.

Temperature and cardiopulmonary parameters remained stable intra-operatively. Rectal temperature ranged between 35.5°C and 38.0°C, heart rate between 74 and 90 beats/minute, respiratory rate between 16 and 24 breaths/minute and blood oxygen saturation between 88% and 98%. The surgery took 80 minutes and the patient recovered spontaneously from anaesthesia 20 minutes later with no untoward effects. Limb paralysis extended for 5 hours post-operatively suggesting prolonged motor and possible sensory blockade.

## Discussion

To the researchers’ knowledge, this is the first reported case of the use of brachial plexus blockade to supplement general anaesthesia to facilitate forelimb surgery in an adult cheetah. Several anaesthesia protocols, for various manipulations in cheetahs, have been reported. They include alphaxalone-alphadolone [11]; tiletamine- zolazepam-medetomidine [12]; medetomidine-ketamine-isoflurane [13]; tiletamine-zolazepam, ketamine, and xylazine [14]; medetomidine or midazolam in combination with ketamine or tiletamine/zolazepam [15]; tiletamine-zolazepam [16].

Local anaesthetics have been clinically used as adjuncts to light general anesthesia in both small and large animals and are reported to have a unique ability to block the sensation of pain [10,17]. A volume of 0.3 mL kg-1 of 2% lidocaine has been reported to be adequate for brachial plexus block in dogs [6]. In this study, 20 mls of 2% lidocaine was used to perform the brachial plexus block on the cheetah weighing 65 kgs, with a successful outcome. It is important to note that the total volume of local anesthetic solution injected plays an important role in the effectiveness and success of brachial plexus block [2]. In the current case, we report excellent intraoperative and post-operative analgesia despite very deep surgical stimulation elicited by the orthopaedic procedure. Absence of autonomic cardiopulmonary reactions to the surgical manipulation may be attributed to the efficacy of brachial plexus block. Apart from lidocaine, other local anaesthetics can be used; bupivacaine has been shown to confer long term perioperative analgesia while rupivacaine provides superior analgesia with minimal motor effects [18] owing to the fact that it is less potent at blocking Aβ fibres, but more potent in blocking Aδ and C fibres [19].

Lidocaine has been reported to possess fast onset and intermediate duration of action of up to 2 hours [17]. The explanation for prolonged limb paralysis, as observed through knuckled carpus and limb dragging while walking, may be attributed, in addition to anaesthetic effect of lignocaine, the protracted trauma to peripheral nerves at the time of accident, direct injury to brachial nerves by the needle during local block and excessive stretching of the nerves during surgical manipulation [20].

Perioperative analgesic protocol has an impact on patient well-being that often extends far beyond the immediate anaesthetic period [21]. Providing analgesia to wild felids is essential because, in addition to managing pain, it also quickens recovery and healing, prevents self-mutilation and permits an earlier return to feeding [22]. Absence of post-operative complications in the current case may be attributed to adequate perioperative analgesia and antibiotic cover.

The maintenance of stable blood oxygen saturation was suggestive of well maintained cardiac and respiratory functions. On the other hand, it is speculated that the mild hypothermia observed in the current case may have been due to the effect of ambient temperature, the action of the anaesthetic drugs and anaesthetic adjuncts, pre-surgical preparation of the surgical site with cold preparation liquids and, the prolonged and invasive nature of the surgical procedure.

The patient in this report was considered an ASA (American Society of Anaesthesiologists) Class III anesthetic risk as indicated by leukocytosis, dehydration and history of untreated pain. It is known that patients that suffer untreated pain for a prolonged period pose a major anaesthetic risk intra-operatively as high doses of anaesthetic drugs are required to produce a surgical plane of anaesthesia, when compared to those whose pain is well managed prior to surgery [21]. A multimodal approach to anaesthesia with concurrent fluid therapy were therefore considered appropriate for the management of this patient.

## Conclusion

The use of brachial plexus block with a light plane of general anaesthesia proved successful. This protocol provided highly effective analgesia, with a fast onset and longer duration analgesia. Brachial plexus block had a sparing effect on isoflurane anaesthesia as evidenced by the concentration used for maintenance of anaesthesia and the stability of the cardiopulmonary function. Moreover, absence of autonomic cardiopulmonary reactions to the surgical manipulation may be attributed to the efficacy of brachial plexus block. Recovery was smooth and uneventful. This anaesthesia protocol is therefore recommended for surgeries of the forelimb in wild cats.

## Consent

The consent was obtained from the Kenya Wildlife Service for the management and publication of this case report and any accompanying images.

### Endnotes

^a^Domitor®; S5 Veterinary Medicine; Novartis South Africa Ltd. ^b^Ketalar 50 mg/ml, Pfizer Inc, New York, USA. ^c^Lidocaine, Mac Pharmaceuticals Limited, Nairobi-Kenya. ^d^Furane, Baxter, Austria. ^e^Betamox® Norbrook veterinary Pharmaceuticals, Nairobi-Kenya ^f^Rimadyl®; Pfizer corporation Austria GmbH, Austria.
